# Supplementary material for: Preclinical Evaluation of the Safety, Toxicity and Efficacy of Genetically Modified Wharton’s Jelly Mesenchymal Stem/Stromal Cells Expressing the Antimicrobial Peptide SE-33
Source: Cells. 2025 Feb 26;14(5):341. doi: 10.3390/cells14050341 (PMC11898551; doi:10.3390/cells14050341)
Supplement: Supplementary file 1 [file cells-14-00341-s001.zip › cells-3452206-supplementary.pdf]

Supplementary Materials

Article

Preclinical Evaluation of the Safety, Toxicity and Efficacy of Genetically Modified Wharton's Jelly Mesenchymal Stem/Stromal Cells Expressing the Antimicrobial Peptide SE-33

Vagif Ali oglu Gasanov [1,*], Dmitry Alexandrovich Kashirskikh [1], Victoria Alexandrovna Khotina [1,*], Daria Mikhailovna Kuzmina [2], Sofya Yurievna Nikitochkina [1], Irina Vasilievna Mukhina [2], Ekaterina Andreevna Vorotelyak [1,3] and Andrey Valentinovich Vasiliev [1]

[1] Koltzov Institute of Developmental Biology of Russian Academy of Sciences, Moscow 119334, Russia; dim.kashirsckih@gmail.com (D.A.K.); nsophie.0107@gmail.com (S.Y.N.); vorotelyak@yandex.ru (E.A.V.); 113162@bk.ru (A.V.V.)
[2] Department of Normal Physiology, Privolzhsky Research Medical University of Ministry of Health of the Russian Federation, Nizhny Novgorod 603005, Russia; dariak2294@gmail.com (D.M.K.); mukhinaiv@mail.ru (I.V.M.)
[3] Department of Cell Biology, Biological Faculty, Lomonosov Moscow State University, Moscow 119234, Russia
* Correspondence: gasanovvagif@gmail.com (V.A.o.G.); v.a.khotina.bio@gmail.com (V.A.K.)

Table S1. Body weight (g) of mice following repeated intravenous administration of genetically modified MSCs expressing SE-33 (M ± SEM)

Observation period	Control (0,9% NaCl)		Control (WJ-MSC 2,5×10^7 cells/kg)		WJ-MSC-SE33					
					0,5×10^7 cells/kg		1,25×10^7 cells/kg		2,5×10^7 cells/kg	
	Male (n=24)	Female (n=24)	Male (n=24)	Female (n=24)	Male (n=24)	Female (n=24)	Male (n=24)	Female (n=24)	Male (n=24)	Female (n=24)
0 day	18,90±0,16	18,67±0,14	18,92±0,15	18,75±0,15	18,85±0,16	18,67±0,15	18,94±0,17	18,79±0,15	18,85±0,17	18,69±0,15
7 day	19,00±0,14	18,88±0,15	19,31±0,16	19,02±0,15	19,13±0,16	19,10±0,16	19,27±0,18	19,19±0,14	19,21±0,15	19,08±0,15
14 day	19,31±0,15	19,21±0,14	19,67±0,14	19,35±0,16	19,52±0,16	19,44±0,15	19,48±0,16	19,60±0,12	19,67±0,14	19,44±0,12
28 day	20,21±0,26	20,17±0,23	20,42±0,22	20,29±0,19	20,50±0,23	20,42±0,28	20,67±0,31	20,46±0,19	20,63±0,24	20,33±0,14

Table S2. Rectal temperature (°C) of mice following repeated intravenous administration of genetically modified MSCs expressing SE-33 (mean ± SEM)

Observation period	Control (0,9% NaCl)		Control (WJ-MSC 2,5×10^7 cells/kg)		WJ-MSC-SE33					
					0,5×10^7 cells/kg		1,25×10^7 cells/kg		2,5×10^7 cells/kg	
	Male (n=24)	Female (n=24)	Male (n=24)	Female (n=24)	Male (n=24)	Female (n=24)	Male (n=24)	Female (n=24)	Male (n=24)	Female (n=24)
0 day	37,58±0,04	37,59±0,05	37,6±0,04	37,64±0,03	37,66±0,04	37,64±0,04	37,64±0,04	37,58±0,05	37,64±0,04	37,63±0,04
7 day	37,59±0,04	37,55±0,05	37,65±0,04	37,59±0,04	37,59±0,03	37,63±0,04	37,58±0,04	37,64±0,04	37,60±0,05	37,59±0,04
14 day	37,62±0,04	37,65±0,04	37,64±0,04	37,62±0,04	37,63±0,04	37,6±0,04	37,63±0,04	37,58±0,03	37,63±0,04	37,63±0,04
28 day	37,59±0,04	37,62±0,04	37,60±0,06	37,64±0,06	37,61±0,06	37,59±0,06	37,59±0,04	37,60±0,06	37,59±0,06	37,63±0,05

Table S3. Effect of repeated intravenous administration of WJ-MSC-SE33 on the behavioral parameters of mice in the open field test (M±SEM)

Observation period	Control (0,9% NaCl)		Control (WJ-MSC 2,5×10^7 cells/kg)		WJ-MSC-SE33					
					0,5×10^7 cells/kg		1,25×10^7 cells/kg		2,5×10^7 cells/kg	
	Male (n=24)	Female (n=24)	Male (n=24)	Female (n=24)	Male (n=24)	Female (n=24)	Male (n=24)	Female (n=24)	Male (n=24)	Female (n=24)
Horizontal activity (duration, sec)										
0 day	212,01±3,79	201,12±4,03	212,25±3,53	209,46±3,4	212,25±3,53	206,39±3,78	206,97±3,68	209,43±4,1	205,83±3,08	204,40±3,74

14 day	205,6±3,94	204,48±4,27	203,04±3,69	202,65±3,57	203,07±3,25	206,1±3,93	205,95±4,02	208,63±3,32	208,86±3,96	202,8±3,71
28 day	205,24±5,76	212,45±5,47	202,46±5,49	203,50±5,93	207,80±5,62	210,03±5,55	208,10±5,18	202,49±6,13	206,97±6,16	210,67±6,98
Vertical activity (number of vertical stands)										
0 day	28,21±0,37	27,79±0,34	28,25±0,34	28,96±0,33	28,33±0,33	28,33±0,35	28,63±0,31	28,04±0,36	28,17±0,34	28,63±0,32
14 day	28,04±0,38	28,42±0,37	28,67±0,37	27,88±0,33	28,75±0,33	29,17±0,36	29,04±0,27	28,46±0,34	28,79±0,35	28,75±0,36
28 day	28,30±0,58	27,50±0,50	28,80±0,47	28,00±0,68	28,20±0,59	28,60±0,58	28,50±0,58	28,30±0,52	28,60±0,54	28,70±0,56
Grooming duration, sec										
0 day	3,25±0,04	3,27±0,04	3,24±0,05	3,27±0,04	3,23±0,04	3,24±0,04	3,27±0,04	3,21±0,04	3,24±0,04	3,28±0,04
14 day	3,27±0,04	3,29±0,04	3,27±0,04	3,30±0,04	3,33±0,04	3,29±0,04	3,28±0,04	3,25±0,05	3,34±0,04	3,32±0,04
28 day	3,27±0,06	3,26±0,07	3,30±0,04	3,39±0,07	3,29±0,06	3,25±0,07	3,29±0,07	3,17±0,06	3,32±0,06	3,27±0,04
Duration of freezing reaction, sec										
0 day	0,62±0,03	0,67±0,02	0,65±0,03	0,61±0,03	0,68±0,02	0,63±0,03	0,64±0,03	0,66±0,03	0,65±0,03	0,62±0,03
14 day	0,65±0,03	0,64±0,03	0,66±0,03	0,67±0,02	0,67±0,02	0,65±0,03	0,64±0,03	0,61±0,03	0,64±0,03	0,63±0,03
28 day	0,61±0,04	0,68±0,04	0,63±0,05	0,66±0,04	0,64±0,03	0,66±0,05	0,63±0,03	0,64±0,05	0,62±0,04	0,64±0,04
Duration of sniffing reaction, sec										
0 day	44,84±1,76	45,19±1,7	47,06±1,65	46,12±1,99	44,08±1,65	46,2±1,68	47,9±1,65	45,85±1,73	48,15±1,97	46,34±1,66
14 day	46,58±1,68	49,81±1,79	49,72±1,8	49,09±1,65	46,13±1,99	47,46±2,35	43,48±1,85	46,82±1,91	48,04±2	48,36±1,41
28 day	47,15±2,32	45,32±3,11	48,78±2,60	43,83±2,75	47,56±2,61	48,28±2,47	46,96±2,98	46,53±2,24	46,10±2,01	44,48±2,28

Table S4. Effect of repeated intravenous WJ-MSC-SE33 administration on the morphological composition of the blood in mice (M±SEM)

Observation period	Control (0,9% NaCl)		Control (WJ-MSC 2,5×10^7 cells/kg)		WJ-MSC-SE33					
					0,5×10^7 cells/kg		1,25×10^7 cells/kg		2,5×10^7 cells/kg	
	Male (n=6)	Female (n=6)	Male (n=6)	Female (n=6)	Male (n=6)	Female (n=6)	Male (n=6)	Female (n=6)	Male (n=6)	Female (n=6)
Hemoglobin, g/L										
15 day	146,33±2,16	144,50±3,91	148,67±2,76	149,67±2,75	147,00±1,37	145,33±2,09	143,00±3,12	144,00±1,71	143,00±4,03	142,67±4,12
30 day	145,83±2,29	147,33±3,36	144,17±2,24	143,00±3,38	147,50±2,46	148,17±3,00	144,67±3,36	151,67±2,50	145,33±3,13	144,67±3,87
Erythrocytes, ×10^{12}/L										
15 day	8,50±0,14	8,33±0,28	8,85±0,22	8,88±0,13	8,78±0,15	8,65±0,15	8,45±0,11	8,60±0,11	8,51±0,23	8,47±0,27
30 day	8,38±0,17	8,41±0,23	8,55±0,13	8,30±0,46	8,82±0,19	8,78±0,20	8,51±0,20	8,98±0,15	8,72±0,18	8,65±0,24
Leukocytes, ×10^9/L										

15 day	7,98±0,87	7,05±0,79	6,72±0,98	5,93±0,98	6,23±0,65	5,88±0,76	6,03±0,88	5,67±0,98	6,53±0,91	4,98±0,73
30 day	7,77±0,50	6,88±0,87	**3,17±0,54***	**2,83±0,58***	**3,63±0,46***	**3,00±0,42***	**2,98±0,43***	**2,50±0,29***	**2,52±0,43***	**2,30±0,52***
Platelets, $\times 10^9$/L										
15 day	641,83±44,93	629,33±44,83	628,50±77,86	680,00±60,69	607,17±29,67	583,17±32,94	709,00±48,48	612,17±56,52	635,17±40,12	679,33±60,19
30 day	656,83±48,41	658,17±32,43	587,33±60,05	649,17±53,17	626,33±35,18	628,00±23,89	616,50±53,88	624,17±50,68	600,00±33,64	604,33±53,56
ESR, mm/h										
15 day	1,17±0,17	1,33±0,21	1,33±0,33	1,33±0,21	1,17±0,17	1,33±0,33	1,17±0,17	1,33±0,21	1,17±0,17	1,17±0,17
30 day	1,33±0,21	1,17±0,17	1,17±0,17	1,17±0,17	1,33±0,21	1,17±0,17	1,50±0,34	1,33±0,33	1,33±0,33	0,17±0,17

Table S5. Effect of repeated intravenous WJ-MSC-SE33 administration on leukocyte count in mice (M±SEM)

Observation period	Control (0,9% NaCl)		Control (WJ-MSC 2,5×10^7 cells/kg)		WJ-MSC-SE33					
					0,5×10^7 cells/kg		1,25×10^7 cells/kg		2,5×10^7 cells/kg	
	Male (n=6)	Female (n=6)	Male (n=6)	Female (n=6)	Male (n=6)	Female (n=6)	Male (n=6)	Female (n=6)	Male (n=6)	Female (n=6)
Band neutrophils, %										
15 day	0,17±0,17	0	0,17±0,17	0,17±0,17	0	0,17±0,17	0	0	0,17±0,17	0
30 day	0	0	0	0	0,17±0,17	0	0	0	0,17±0,17	0
Segmented neutrophils, %										
15 day	17,50±1,48	17,17±1,40	18,67±2,82	19,17±2,33	19,00±1,53	20,83±2,96	19,17±1,78	18,50±2,05	20,50±2,74	19,83±1,78
30 day	18,83±1,14	19,67±1,23	21,50±1,09	21,17±2,04	21,00±2,57	22,50±1,73	23,83±2,56	21,33±1,78	23,33±2,47	23,83±2,01
Eosinophils, %										
15 day	2,00±0,77	2,17±0,60	2,33±0,49	2,00±0,73	2,17±0,48	2,33±0,71	2,17±0,65	2,50±0,72	2,67±0,49	2,50±0,85
30 day	2,33±0,67	2,83±0,60	1,17±0,48	1,33±0,49	1,50±0,43	1,67±0,42	1,50±0,56	1,83±0,48	1,50±0,56	1,33±0,49
Basophils, %										
15 day	0	0	0	0	0	0	0	0	0	0
30 day	0	0	0	0	0	0	0	0	0	0
Monocytes, %										
15 day	3,00±0,73	3,50±0,76	3,67±0,71	3,00±0,52	3,50±0,43	3,83±0,70	3,33±0,67	2,67±0,71	2,33±0,80	3,17±0,60
30 day	3,17±0,60	3,00±0,73	2,50±0,67	2,17±0,87	3,00±0,58	3,17±0,60	2,83±0,79	3,17±0,60	2,50±0,67	2,17±0,83
Lymphocytes, %										
15 day	77,33±2,32	77,17±1,74	75,17±3,24	75,67±2,38	75,33±1,63	72,83±2,93	75,33±2,14	76,33±2,39	74,33±2,51	74,50±2,45
30 day	75,67±1,31	74,50±1,38	74,83±1,25	75,33±3,13	74,33±2,82	72,67±1,61	71,83±2,98	73,67±1,86	72,50±2,59	72,67±2,36

Table S6. Effect of repeated intravenous WJ-MSC-SE33 administration of on the blood biochemical parameters in mice (M±SEM)

Observation period	Control (0,9% NaCl)		Control (WJ-MSC 2,5×10⁷ cells/kg)		WJ-MSC-SE33					
					0,5×10⁷ cells/kg		1,25×10⁷ cells/kg		2,5×10⁷ cells/kg	
	Male (n=6)	Female (n=6)	Male (n=6)	Female (n=6)	Male (n=6)	Female (n=6)	Male (n=6)	Female (n=6)	Male (n=6)	Female (n=6)
Total protein, g/L										
15 day	57,33±1,51	57,01±1,98	54,71±0,94	57,20±0,87	55,42±0,84	57,40±1,00	56,49±0,67	57,53±1,08	56,11±2,10	57,40±2,06
30 day	57,12±2,35	57,89±1,18	57,32±1,71	56,97±1,84	57,29±1,56	58,13±2,87	58,45±2,23	59,16±1,85	57,02±1,34	58,10±2,19
Albumin, g/L										
15 day	32,06±1,43	34,02±1,45	32,74±0,63	33,61±0,96	32,94±1,02	34,06±1,00	33,06±0,70	33,57±0,86	32,98±2,05	34,27±1,82
30 day	34,41±1,97	34,49±1,40	33,69±1,51	34,83±1,64	33,66±1,69	34,42±1,56	32,54±0,98	34,96±1,96	34,11±1,54	34,21±1,93
Urea, mmol/L										
15 day	6,64±0,09	6,63±0,09	6,61±0,08	6,67±0,10	6,62±0,12	6,64±0,09	6,67±0,09	6,60±0,09	6,59±0,09	6,61±0,10
30 day	6,63±0,10	6,60±0,12	6,62±0,09	6,65±0,09	6,61±0,08	6,67±0,12	6,65±0,10	6,58±0,10	6,60±0,09	6,62±0,12
Creatinine, μmol/L										
15 day	74,77±0,79	73,65±0,90	74,39±0,94	74,28±0,79	74,16±1,62	73,72±1,19	74,51±1,05	74,08±1,17	74,12±0,89	74,46±0,82
30 day	74,17±1,19	74,05±0,99	74,36±0,98	74,41±0,72	73,74±0,94	74,60±1,30	74,49±0,90	74,62±1,08	74,28±1,00	74,32±1,08
Total cholesterol, mmol/L										
15 day	2,29±0,27	2,00±0,17	2,30±0,15	1,97±0,14	2,25±0,15	2,03±0,07	2,29±0,20	1,99±0,11	2,05±0,18	2,01±0,19
30 day	2,02±0,26	2,01±0,22	1,95±0,22	1,87±0,23	1,88±0,26	1,82±0,19	1,90±0,23	1,78±0,32	1,98±0,21	1,89±0,29
Triglycerides, mmol/L										
15 day	1,02±0,09	1,04±0,08	1,08±0,11	0,99±0,10	1,05±0,09	1,09±0,10	1,10±0,10	1,06±0,12	1,10±0,12	1,13±0,16
30 day	1,03±0,16	1,01±0,14	1,05±0,13	1,10±0,12	1,07±0,12	1,00±0,13	1,01±0,11	1,06±0,09	1,03±0,11	1,00±0,14
Glucose, mmol/L										
15 day	4,56±0,10	4,52±0,09	4,50±0,11	4,57±0,12	4,53±0,09	4,59±0,09	4,62±0,09	4,51±0,11	4,50±0,10	4,60±0,08
30 day	4,56±0,06	4,61±0,07	4,48±0,07	4,59±0,09	4,55±0,10	4,59±0,09	4,60±0,11	4,54±0,12	4,58±0,10	4,51±0,08
Aspartate aminotransferase, U/L										
15 day	193,79±31,34	159,38±18,06	205,74±20,11	185,14±13,95	198,65±18,95	188,34±20,13	196,59±27,90	163,76±20,18	201,78±28,59	186,46±25,71
30 day	184,28±30,06	190,61±23,08	220,26±39,97	207,33±25,09	203,45±32,09	214,10±27,41	190,25±37,72	180,66±31,84	199,13±36,00	190,79±34,77
Alanine aminotransferase, U/L										

15 day	54,80±8,61	45,44±5,90	46,59±4,45	42,34±3,79	45,68±4,48	41,27±5,58	50,80±5,06	41,32±5,08	53,01±6,08	45,15±6,27
30 day	51,49±7,48	45,62±6,85	40,40±5,06	41,18±4,42	42,23±5,21	47,09±4,33	39,95±6,33	42,63±5,59	44,88±10,13	47,16±8,40
Alkaline phosphatase, U/L										
15 day	128,78±15,21	120,90±16,34	123,92±15,30	143,82±7,00	148,83±12,96	140,50±6,82	141,06±8,13	137,63±9,71	124,05±17,72	142,45±13,16
30 day	127,01±9,72	124,16±12,34	125,40±12,29	132,34±9,38	136,83±10,71	142,16±11,29	139,11±11,71	138,82±14,09	133,89±14,55	144,14±16,05
Total bilirubin, μmol/L										
15 day	1,71±0,52	2,05±0,39	1,80±0,63	1,98±0,53	1,95±0,55	2,10±0,39	2,14±0,31	2,32±0,59	1,92±0,58	1,90±0,47
30 day	1,97±0,42	1,88±0,33	1,53±0,47	1,76±0,41	2,07±0,28	1,81±0,40	1,77±0,51	1,84±0,42	2,10±0,40	1,93±0,40
Calcium, mmol/L										
15 day	2,31±0,08	2,33±0,06	2,27±0,09	2,29±0,07	2,34±0,06	2,30±0,06	2,33±0,06	2,31±0,06	2,28±0,06	2,30±0,07
30 day	2,32±0,05	2,31±0,09	2,34±0,07	2,32±0,06	2,27±0,07	2,31±0,06	2,35±0,08	2,29±0,08	2,34±0,07	2,27±0,06
Potassium, mmol/L										
15 day	6,58±0,12	6,56±0,11	6,48±0,13	6,62±0,12	6,51±0,10	6,54±0,10	6,58±0,14	6,50±0,11	6,59±0,10	6,60±0,13
30 day	6,56±0,13	6,55±0,12	6,54±0,09	6,52±0,09	6,56±0,11	6,49±0,08	6,53±0,11	6,52±0,09	6,49±0,09	6,55±0,11
Sodium, mmol/L										
15 day	139,17±0,46	139,28±0,58	139,34±0,55	139,30±0,54	139,72±0,61	139,12±0,58	139,37±0,65	138,95±0,56	139,14±0,45	138,90±0,49
30 day	138,93±0,44	139,43±0,55	139,27±0,60	139,01±0,54	139,24±0,64	138,91±0,50	138,84±0,50	139,27±0,59	139,10±0,51	139,24±0,60

Table S7. Effect of repeated intravenous WJ-MSC-SE33 administration on urinalysis in mice (M±SEM)

Observation period	Control (0,9% NaCl)		Control (WJ-MSC 2,5×10⁷ cells/kg)		WJ-MSC-SE33					
					0,5×10⁷ cells/kg		1,25×10⁷ cells/kg		2,5×10⁷ cells/kg	
	Male (n=12)	Female (n=12)	Male (n=12)	Female (n=12)	Male (n=12)	Female (n=12)	Male (n=12)	Female (n=12)	Male (n=12)	Female (n=12)
pH										
14 day	6,42±0,10	6,62±0,11	6,67±0,10	6,54±0,11	6,54±0,13	6,42±0,10	6,60±0,12	6,54±0,10	6,46±0,11	6,58±0,15
28 day	6,54±0,13	6,50±0,14	6,67±0,11	6,30±0,11	6,37±0,11	6,35±0,13	6,42±0,13	6,42±0,12	6,62±0,12	6,58±0,15
Specific gravity, g/ml										
14 day	1,002±0,001	1,003±0,001	1,002±0,001	1,003±0,001	1,003±0,001	1,002±0,001	1,002±0,001	1,002±0,001	1,004±0,001	1,004±0,002
28 day	1,002±0,001	1,002±0,001	1,002±0,001	1,002±0,001	1,003±0,001	1,001±0,002	1,003±0,001	1,002±0,001	1,001±0,001	1,003±0,002
Leukocytes, number per field of view										
14 day	0-0	1-0	1-0	0-1	1-0	0-1	1-1	0-0	0-1	1-1
28 day	0-0	0-1	1-1	1-1	0-0	0-1	1-1	1-1	0-1	1-1

Erythrocytes, number per field of view										
14 day	0-0	1-1	1-0	1-1	1-0	1-1	1-1	0-0	1-1	0-1
28 day	0-1	1-1	1-0	1-0	1-1	1-1	0-0	1-1	1-1	0-1
Glucose, mmol/L										
14 day	negative	negative	negative	negative	negative	negative	negative	negative	negative	negative
28 day	negative	negative	negative	negative	negative	negative	negative	negative	negative	negative
Ketone bodies, mmol/L										
14 day	negative	negative	negative	negative	negative	negative	negative	negative	negative	negative
28 day	negative	negative	negative	negative	negative	negative	negative	negative	negative	negative
Bilirubin, μmol/L										
14 day	negative	negative	negative	negative	negative	negative	negative	negative	negative	negative
28 day	negative	negative	negative	negative	negative	negative	negative	negative	negative	negative
Total protein, g/l										
14 day	0,03±0,03	0,50±0,03	0,03±0,03	0,00±0,00	0,05±0,03	0,05±0,03	0,03±0,03	0,05±0,03	0,05±0,03	0,05±0,03
28 day	0,03±0,03	0,03±0,03	0,05±0,03	0,05±0,05	0,05±0,03	0,05±0,03	0,05±0,03	0,00±0,03	0,05±0,03	0,03±0,03

Table S8. Effect of repeated intravenous WJ-MSC-SE33 administration on organ mass coefficients in mice (M±SEM)

Observation period	Control (0,9% NaCl)		Control (WJ-MSC 2,5×10⁷ cells/kg)		WJ-MSC-SE33					
					$0,5 \times 10^7$ cells/kg		$1,25 \times 10^7$ cells/kg		$2,5 \times 10^7$ cells/kg	
	Male (n=12)	Female (n=12)	Male (n=12)	Female (n=12)	Male (n=12)	Female (n=12)	Male (n=12)	Female (n=12)	Male (n=12)	Female (n=12)
Heart										
15 day	4,81±0,17	4,91±0,18	4,77±0,16	4,77±0,14	4,98±0,14	4,90±0,18	4,73±0,18	4,74±0,17	4,97±0,14	4,88±0,16
30 day	4,62±0,11	4,71±0,15	4,59±0,13	4,61±0,16	4,51±0,15	4,78±0,16	4,56±0,16	4,75±0,15	4,60±0,17	4,64 ±0,15
Lungs (both)										
15 day	9,40±0,24	9,22±0,22	9,50±0,29	9,41±0,26	9,38±0,31	9,47±0,30	9,39±0,31	9,49±0,27	9,30±0,33	9,11±0,26
30 day	9,05±0,33	8,94±0,36	9,18±0,30	9,09±0,31	9,39±0,33	9,29±0,20	8,97±0,32	8,97±0,35	9,30±0,30	9,11±0,27
Thymus										
15 day	3,59±0,09	3,78±0,06	3,55±0,09	3,67±0,10	3,65±0,09	3,67±0,09	3,61±0,10	3,74±0,10	3,55±0,09	3,73±0,11
30 day	3,41±0,06	3,42±0,10	3,41±0,10	3,42±0,10	3,44±0,11	3,41±0,09	3,47±0,05	3,47±0,08	3,44±0,07	3,41±0,08
Liver										
15 day	58,63±1,08	59,08±0,62	57,55±0,75	58,81±1,05	58,75±0,94	59,36±1,01	58,33±0,79	58,02±1,05	57,70±1,20	58,40±1,03

30 day	55,13±0,98	56,30±1,15	56,46±0,95	56,48±0,99	55,46±1,07	55,29±0,86	55,64±1,20	55,90±0,94	55,69±0,85	56,46±0,91
Spleen										
15 day	6,19±0,13	6,18±0,08	6,17±0,14	6,09±0,13	6,29±0,12	6,24±0,14	6,30±0,13	6,17±0,11	6,10±0,14	6,19±0,15
30 day	5,85±0,15	5,87±0,13	5,75±0,09	5,98±0,14	5,87±0,16	5,94±0,15	5,86±0,13	6,06±0,12	5,78±0,13	5,97±0,09
Kidneys (both)										
15 day	12,50±0,30	12,25±0,24	12,58±0,29	12,05±0,25	12,25±0,22	12,34±0,26	12,35±0,21	12,50±0,29	12,15±0,29	12,40±0,29
30 day	11,80±0,36	11,97±0,26	11,54±0,29	11,99±0,30	11,88±0,32	11,94±0,35	11,70±0,30	12,19±0,21	11,92±0,21	12,06±0,18
Brain										
15 day	20,51±0,32	20,28±0,24	20,31±0,32	19,97±0,26	20,44±0,27	20,41±0,28	20,34±0,18	20,36±0,18	20,26±0,31	20,26±0,30
30 day	19,53±0,24	19,89±0,21	19,59±0,32	19,75±0,29	19,40±0,28	19,87±0,32	19,24±0,36	19,78±0,26	19,46±0,31	19,87±0,14
